# LED Photobiomodulation at 630 nm Attenuates TNF-α-Induced Senescence-Associated Responses in Association with Mitochondrial Homeostasis

**DOI:** 10.3390/ijms27219784

**Published:** 2026-10-30

**Authors:** Xueqian Li, Lihong Zhang, Nan Wang, Xiao Yang, Wenting Xie, Qingtai Meng, Longfei Hu, Hailiang Liu, Wuqi Song

**Affiliations:** Heilongjiang Key Laboratory of Immunity and Infection, Department of Microbiology, Wu Lien-Teh Institute, School of Basic Medical Sciences, Harbin Medical University, Harbin 150081, China

**Keywords:** inflammation, SASP, LED photobiomodulation, mitochondrial homeostasis, autophagy-related proteins

## Abstract

Chronic inflammation can accelerate the aging of joint tissues, characterized by cellular senescence, inflammatory degeneration, and disrupted tissue homeostasis. The accumulation of senescent cells and the excessive release of senescence-associated secretory phenotype (SASP) factors are accompanied by mitochondrial dysfunction, including increased oxidative stress, reduced mitochondrial membrane potential, and impaired mitochondrial quality control. Since TNF-α is a key inflammatory mediator in arthritic tissues, we used TNF-α-stimulated human fibroblast-like synoviocytes (MH7A) and chondrocytes (C28/I2), along with hTNF-α transgenic mice, to investigate whether 630 nm LED photobiomodulation therapy (PBMT) attenuates inflammatory and senescence-associated responses and whether these effects are linked to the restoration of mitochondrial homeostasis. Transcriptomic analysis, cell-based assays, and tissue immunohistochemistry were used to assess inflammatory and senescence-associated gene expression, mitochondrial features, autophagy-related proteins, and SIRT1/PGC-1α levels. PBMT reduced the TNF-α-induced expression of IL-6, IL-1β, IL-8, MMP-3, MMP-13, p16, and p21 in both cell models. Further experiments in C28/I2 cells showed that TNF-α increased reactive oxygen species (ROS) production, reduced mitochondrial membrane potential, disrupted mitochondrial ultrastructure, altered autophagy-related protein expression, and decreased SIRT1 and PGC-1α expression. These alterations were partially reversed by PBMT, accompanied by the restoration of mitochondrial homeostasis-related markers. Extending the in vitro findings, in vivo experiments using hTNF-α transgenic mice showed that PBMT enhanced the immunostaining signals of SIRT1 and PGC-1α in joint tissues. Overall, these findings indicate that PBMT attenuates TNF-α-induced inflammatory and senescence-associated responses in joint cells, which are associated with mitochondrial homeostasis, supporting further mechanistic and functional evaluation of this intervention.

## 1. Introduction

Photobiomodulation therapy (PBMT) is a non-pharmacological, non-thermal physical intervention that uses red or near-infrared light to regulate cellular responses in tissues under inflammatory or degenerative stress. A primary proposed mechanism of PBMT involves mitochondrial light responses, in which light at specific wavelengths affects photoreceptors associated with the mitochondrial respiratory chain. This action regulates electron transport, ATP production, nitric oxide (NO) release, and downstream signaling pathways [1,2]. Therefore, the biological effects of PBMT reflect the regulation of cellular energy metabolism and stress adaptation. In joint tissue cells, mitochondrial dysfunction plays a critical role in linking chronic inflammatory stimulation, oxidative stress, cellular senescence, and matrix catabolism. Abnormalities in mitochondrial biogenesis, mitochondrial dynamics, and mitophagy can impair the ability of cells to maintain homeostasis [3].

Chronic inflammation is a major biological factor that accelerates tissue aging. In joint tissues, the prolonged release of inflammatory signals disrupts the homeostatic balance required to maintain the structural integrity, reparative capacity, and mechanical function of cartilage and synovium. Studies of degenerative and inflammatory joint disorders have demonstrated that this disrupted microenvironment is associated with the accumulation of senescent cells, increased matrix degradation, and diminished regenerative potential [4,5,6,7]. Senescent chondrocytes and synoviocytes not only reflect tissue degeneration, but also aggravate local inflammation and further compromise joint homeostasis through the secretion of pro-inflammatory mediators [6,7]. Consequently, inflammation-induced cellular senescence is a key link between chronic joint inflammation and progressive functional deterioration.

Cellular senescence is a multifaceted stress response characterized by sustained cell-cycle arrest, activation of cell cycle inhibitory mechanisms, and the development of a senescence-associated secretory phenotype (SASP) [4,5]. The accumulation of senescent cells can exacerbate local inflammatory responses and pathological tissue remodeling through SASP-driven paracrine senescence and disruption of extracellular matrix homeostasis [5,6,7,8]. Mitochondrial dysfunction is closely linked to these processes. Specifically, increased mitochondrial oxidative stress, altered membrane potential, compromised mitochondrial quality control, and defective autophagy amplify senescence-associated signaling and sustain inflammatory output [9]. Furthermore, in senescent cells, apoptotic stress can trigger the release of mitochondrial DNA, which activates innate immune signaling and promotes SASP factor secretion, thereby reinforcing a self-sustaining inflammatory loop [10]. In joint tissues, particularly in chondrocytes, disruption of mitochondrial biogenesis and autophagic flux is coupled with catabolic reprogramming and loss of matrix integrity, and the SIRT1/PGC-1α axis serves as a key upstream regulator of mitochondrial biogenesis and autophagy [11,12]. Consistent with this, interventions that enhance SIRT1 activity, improve autophagy, promote mitochondrial biogenesis, and reduce mitochondrial oxidative stress have been shown to attenuate age-related tissue dysfunction [13]. Together, these observations suggest that mitochondrial homeostasis is a critical therapeutic target for mitigating inflammation-associated joint aging.

Preclinical evidence for PBMT in rheumatoid arthritis (RA) and osteoarthritis (OA) has been obtained at both the joint-cell and whole-animal levels. In RA, PBMT reduces TNF-α-induced proliferation, migration, and invasion of fibroblast-like synoviocytes and attenuates synovial inflammation and cartilage degradation in collagen-induced arthritis mice [14]; 630 nm LED irradiation has also been shown to alleviate collagen-induced arthritis through inhibition of NF-κB-mediated MMP production [15]. In OA, LED irradiation of chondrocytes under inflammatory stimulation reduces catabolic mediator expression and preserves matrix components, with corresponding histological and pain-related improvements in destabilization of the medial meniscus models [16]. Similarly, LED irradiation of fibroblast-like synoviocytes attenuates inflammatory mediator expression and improves histological and pain-related outcomes in collagenase-induced osteoarthritis models [17]. More specifically, 630 nm LED irradiation has been shown to upregulate BMP4 in TNF-α-stimulated MH7A cells and in hTNF-α transgenic mice, an effect correlated with histological amelioration of arthritis [18]. However, these studies have focused primarily on inflammatory, proliferative, apoptotic, and catabolic endpoints. Whether 630 nm LED irradiation is associated with changes in mitochondrial homeostasis and senescence-associated programs in joint tissue cells under inflammatory stimulation remains unclear.

In this study, we hypothesized that 630 nm LED PBMT may protect joint tissue cells from inflammation-induced senescence-associated damage in association with attenuation of TNF-α-driven cellular senescence and restoration of mitochondrial homeostasis. Because TNF-α is a critical mediator of chronic joint inflammation, we used TNF-α-driven experimental models to mimic inflammatory stress and examine the relationship between inflammatory stress, senescence-associated responses, and mitochondrial dysfunction [19]. Initially, we assessed whether 630 nm LED attenuated TNF-α-induced inflammatory and senescence-associated responses in joint tissue cells. Subsequently, we examined whether these effects were accompanied by restoration of mitochondrial homeostasis in TNF-α-stimulated joint cells. Finally, we examined SIRT1/PGC-1α signals in joint tissues of hTNF-α transgenic mice to determine whether the cell-level effects of PBMT were reflected in vivo. Through this integrated approach, this study aimed to determine whether 630 nm LED irradiation mitigates TNF-α-driven senescence-associated responses alongside restoration of mitochondrial homeostasis during inflammation-induced joint aging.

## 2. Results

### 2.1. Differential Gene Expression and Functional Enrichment Analyses in Chronically Inflamed Synovial Tissue

**Figure 1:**
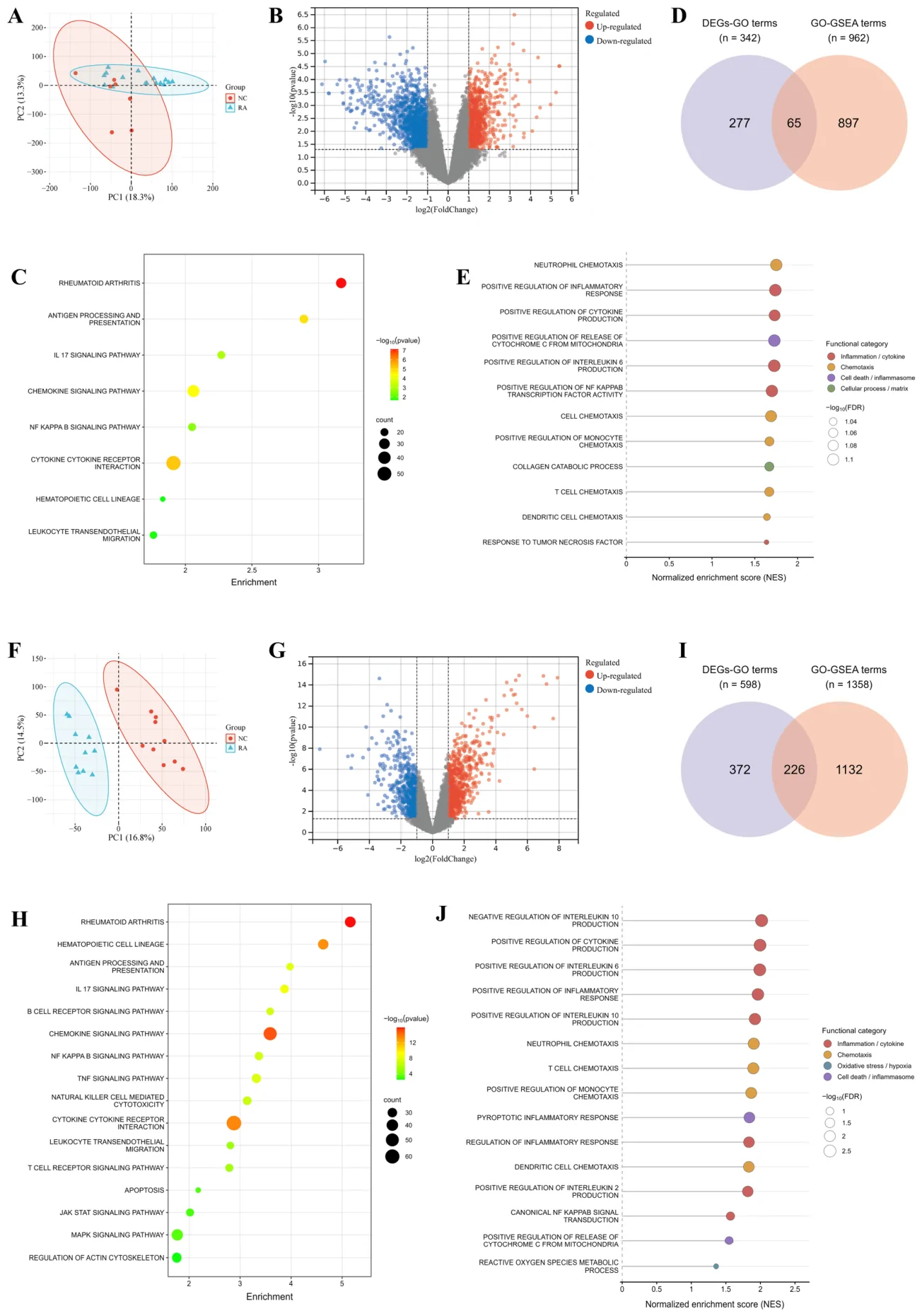
Differential gene expression and functional enrichment analyses in chronically inflamed synovial tissue. (**A**–**E**) Bioinformatic analysis of the GSE77298 dataset comparing chronically inflamed synovial tissues with healthy controls. (**A**) PCA showing sample distribution. (**B**) Volcano plot showing DEGs. (**C**) KEGG enrichment analysis of DEGs. (**D**) Venn diagram showing overlapping GO terms from DEG enrichment and GO-GSEA analysis. (**E**) GSEA of immune-inflammatory, cellular senescence, mitochondrial function-related biological processes. (**F**–**J**) Bioinformatic analysis of the GSE55235 dataset comparing chronically inflamed synovial tissues with healthy controls. (**F**) PCA showing sample distribution. (**G**) Volcano plot showing DEGs. (**H**) KEGG enrichment analysis of DEGs. (**I**) Venn diagram showing overlapping GO terms from DEG enrichment and GO-GSEA analysis. (**J**) GSEA of immune-inflammatory, cellular senescence, mitochondrial function-related biological processes.

To investigate transcriptional alterations related to cellular senescence and mitochondrial dysfunction in chronically inflamed synovial tissues, we analyzed two independent public transcriptomic datasets, GSE77298 and GSE55235. Principal component analysis showed a trend of separation between inflammatory synovial tissue samples and healthy control samples in both datasets. This suggests that under chronic inflammation, synovial tissue has an overall expression profile distinct from that of normal tissue (Figure 1A,F). Using *p* < 0.05 and |log_2_FC| > 1 as screening criteria, differential expression analysis identified 2298 DEGs in GSE77298, including 809 upregulated and 1489 downregulated genes, and 1291 DEGs in GSE55235, including 730 upregulated and 561 downregulated genes (Figure 1B,G). KEGG enrichment analysis showed that DEGs from both datasets were mainly enriched in pathways related to inflammation and immune regulation, indicating a consistent feature of inflammatory and immune activation in chronically inflamed synovial tissues (Figure 1C,H). To further define stable inflammation-related biological processes, we compared GO biological process enrichment based on DEGs with GO-GSEA based on whole-gene ranked lists. Venn diagram analysis identified 65 overlapping biological process terms in GSE77298 and 226 overlapping terms in GSE55235 between the two approaches (Figure 1D,I), suggesting that these shared terms represent robust functional changes in chronic inflammatory tissue. Further GSEA showed that in both datasets, inflammatory synovial tissue samples exhibited positive enrichment for inflammatory response, cytokine production, chemotaxis, and TNF-related biological processes. These changes were accompanied by alterations in ROS metabolism, oxidative stress, and mitochondrial function-related processes (Figure 1E,J). Together, these results suggest that chronic inflammatory tissues are characterized by persistent activation of inflammatory signals and molecular features closely linked to cellular senescence, including oxidative stress and disrupted mitochondrial homeostasis. Based on these findings, TNF-α stimulation was used in subsequent experiments to establish an in vitro inflammatory model with senescence-associated molecular features, in which mitochondrial dysfunction and the molecular responses to LED treatment could be examined.

### 2.2. LED Irradiation at 630 nm Attenuates TNF-α-Induced Inflammatory and Senescence-Associated Responses in MH7A Cells

**Figure 2:**
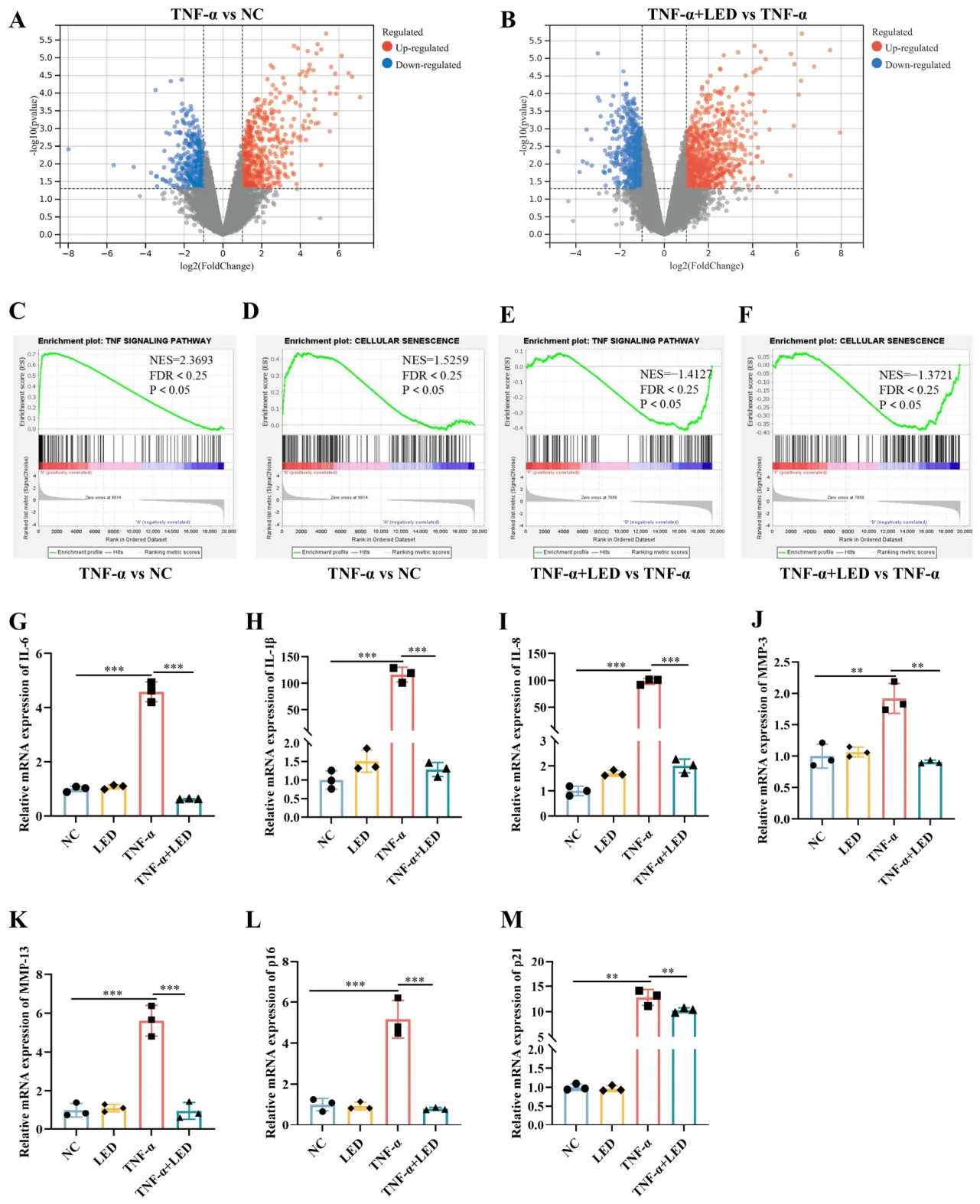
LED irradiation at 630 nm attenuates TNF-α-induced inflammatory and senescence-associated responses in MH7A cells. (**A**,**B**) Volcano plots showing DEGs in TNF-α-stimulated cells and TNF-α + LED-treated cells. Upregulated and downregulated genes are shown in red and blue, respectively. (**C**,**D**) GSEA of TNF signaling and cellular senescence pathways in TNF-α-stimulated cells compared with control cells. (**E**,**F**) GSEA of TNF signaling and cellular senescence pathways in TNF-α + LED-treated cells compared with TNF-α-stimulated cells. (**G**–**M**) RT-qPCR analysis of IL-6, IL-1β, IL-8, MMP-3, MMP-13, p16, and p21 expression. Data are presented as mean ± SD and were analyzed using one-way ANOVA followed by Tukey’s multiple comparisons test. Circles, diamonds, squares, and triangles represent individual data points for the NC, LED, TNF-α, and TNF-α + LED groups, respectively. *n* = 3, ** *p* < 0.01, *** *p* < 0.001.

Based on the enrichment of inflammation and senescence-related pathways in public datasets, we first examined the effect of 630 nm LED irradiation on TNF-α-induced inflammatory responses in fibroblast-like synoviocytes. With *p* < 0.05 and |log_2_FC| > 1 as screening criteria, volcano plot analysis demonstrated that TNF-α stimulation induced significant gene expression changes relative to control cells. A total of 832 DEGs were identified, including 465 upregulated and 367 downregulated genes, indicating that TNF-α successfully induced an inflammatory state in MH7A cells (Figure 2A). In TNF-α-stimulated cells treated with LED, a new differential gene expression profile was observed compared with TNF-α-stimulated cells alone. In total, 1446 DEGs were identified, including 810 upregulated and 636 downregulated genes, suggesting that 630 nm LED irradiation modulated the TNF-α-induced inflammatory response (Figure 2B). GSEA revealed that TNF-α stimulation positively enriched the TNF signaling and cellular senescence gene sets (NES = 2.3693 and 1.5259, respectively; FDR < 0.25 and *p* < 0.05), whereas LED treatment negatively enriched both gene sets (NES = −1.4127 and −1.3721, respectively; FDR < 0.25 and *p* < 0.05) (Figure 2C–F). To validate these transcriptomic results, we performed RT-qPCR analysis of inflammation- and senescence-associated genes. TNF-α significantly increased the expression of IL-6, IL-1β, IL-8, MMP-3, MMP-13, p16, and p21, whereas 630 nm LED irradiation reduced the expression of these genes. LED treatment alone did not induce a clear inflammatory response (Figure 2G–M). These results indicate that 630 nm LED irradiation attenuates TNF-α-induced inflammatory and senescence-associated gene expression in MH7A cells. After confirming this effect in synovial cells, we extended the study to C28/I2 chondrocytes. Because chondrocytes are key effector cells that maintain tissue homeostasis and structural integrity under inflammatory conditions, we next examined whether LED irradiation could reduce TNF-α-induced chondrocyte injury, mitochondrial dysfunction, and senescence-associated phenotypes.

### 2.3. LED Irradiation at 630 nm Attenuates TNF-α-Induced Inflammatory and Senescence-Associated Responses in C28/I2 Cells

**Figure 3:**
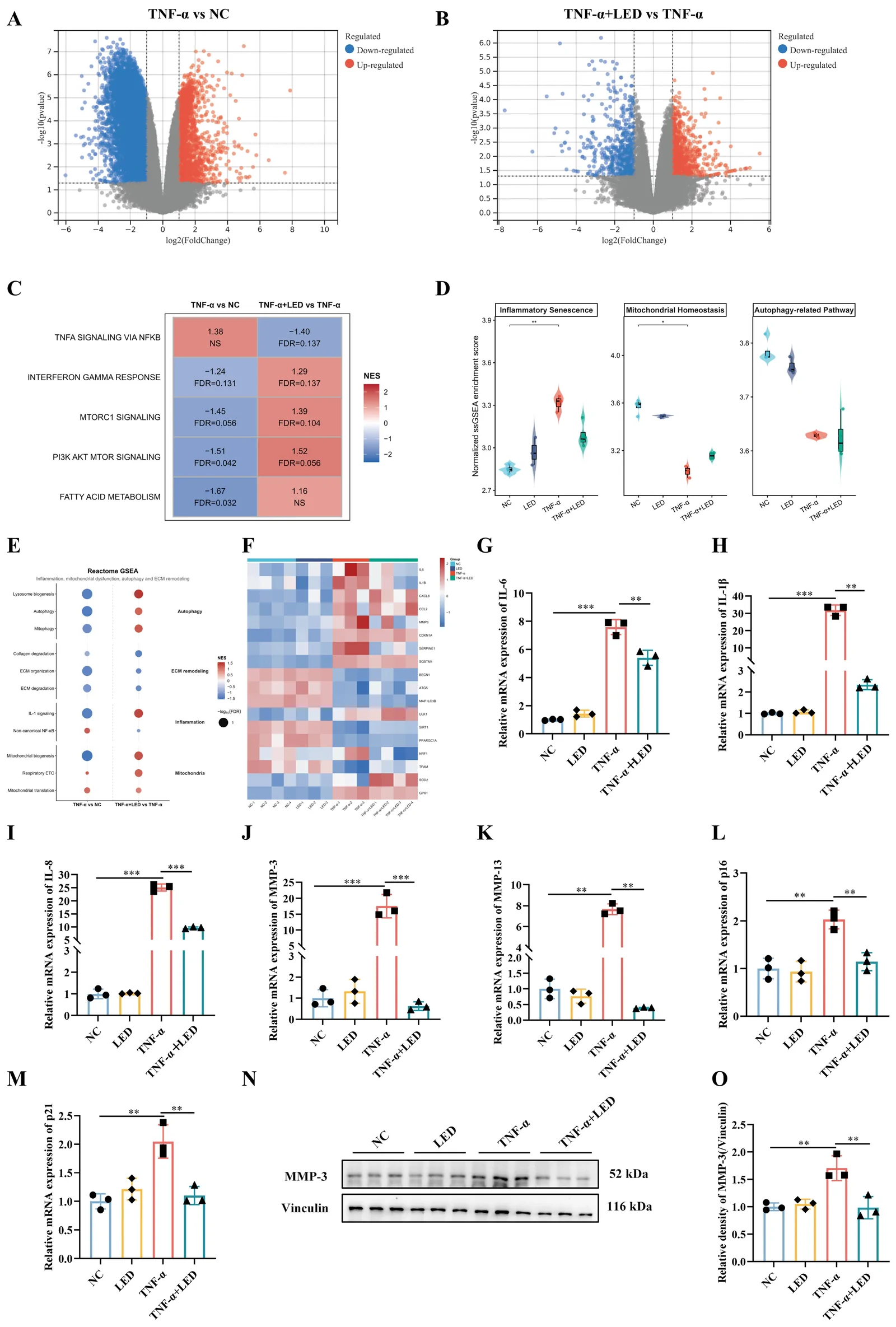
LED irradiation at 630 nm attenuates TNF-α-induced inflammatory and senescence-associated responses in C28/I2 cells. (**A**,**B**) Volcano plots showing DEGs in TNF-α-stimulated cells and TNF-α + LED-treated cells. Upregulated and downregulated genes are shown in red and blue, respectively. (**C**–**E**) GSEA and ssGSEA analyses of inflammatory, senescence-associated, mitochondrial, autophagy-related pathways. In panel (**D**), no significant differences were observed between the TNF-α and TNF-α + LED groups across these pathway scores (*p* > 0.05). (**F**) Heatmap showing representative genes related to autophagy, inflammation, and mitochondrial function. (**G**–**M**) RT-qPCR analysis of IL-6, IL-1β, IL-8, MMP-3, MMP-13, p16, and p21 expression. (**N**,**O**) Representative Western blot and quantification of MMP-3 protein abundance in C28/I2 cells. Vinculin was used as the loading control. Data are presented as mean ± SD and were analyzed using one-way ANOVA followed by Tukey’s multiple comparisons test. Circles, diamonds, squares, and triangles represent individual data points for the NC, LED, TNF-α, and TNF-α + LED groups, respectively. *n* = 3–4, * *p* < 0.05, ** *p* < 0.01, *** *p* < 0.001. NES, normalized enrichment score; FDR, false discovery rate.

Differential gene expression analysis was then performed using sequencing data from C28/I2 chondrocytes, with *p* < 0.05 and |log_2_FC| > 1 as the screening criteria. In the comparison between the TNF-α group and the NC group, 8252 DEGs were identified, including 1996 upregulated and 6256 downregulated genes (Figure 3A). Compared with the TNF-α group, the TNF-α + LED group had 1373 DEGs, including 834 upregulated and 539 downregulated genes (Figure 3B). These results indicate that TNF-α also induced marked transcriptomic changes in C28/I2 cells, whereas LED treatment further altered the gene expression profile after TNF-α stimulation. Unlike the responses observed in MH7A cells, pathway analysis in C28/I2 cells further suggested that TNF-α affected mitochondrial homeostasis, autophagy, and metabolism-related processes. This finding indicates that chondrocytes show more pronounced cellular injury-related features in response to inflammatory stimulation. Pathway scoring and enrichment analyses showed increased inflammatory and senescence-associated signaling after TNF-α treatment, together with abnormalities in mitochondrial function and autophagy-related pathways. Following 630 nm LED irradiation, the inflammatory and mitochondrial showed a partial reversal trend; however, these changes, like the autophagy-related ssGSEA score, did not reach statistical significance (Figure 3C–F). RT-qPCR analysis showed that TNF-α significantly induced the expression of inflammatory cytokines and senescence markers, including IL-6, IL-1β, IL-8, MMP-3, MMP-13, p16, and p21. LED irradiation markedly reduced the TNF-α-induced upregulation of these genes (Figure 3G–M). Western blotting further showed that TNF-α stimulation increased MMP-3 protein abundance in C28/I2 cells, whereas 630 nm LED irradiation significantly reversed this effect (Figure 3N,O). These findings suggest that, in C28/I2 chondrocytes, 630 nm LED irradiation attenuates inflammatory and senescence-associated responses and was accompanied by partial normalization of mitochondrial-function and autophagy-related profiles.

### 2.4. LED Irradiation at 630 nm Restores SIRT1 and PGC-1α Expression in TNF-α-Stimulated C28/I2 Cells

**Figure 4:**
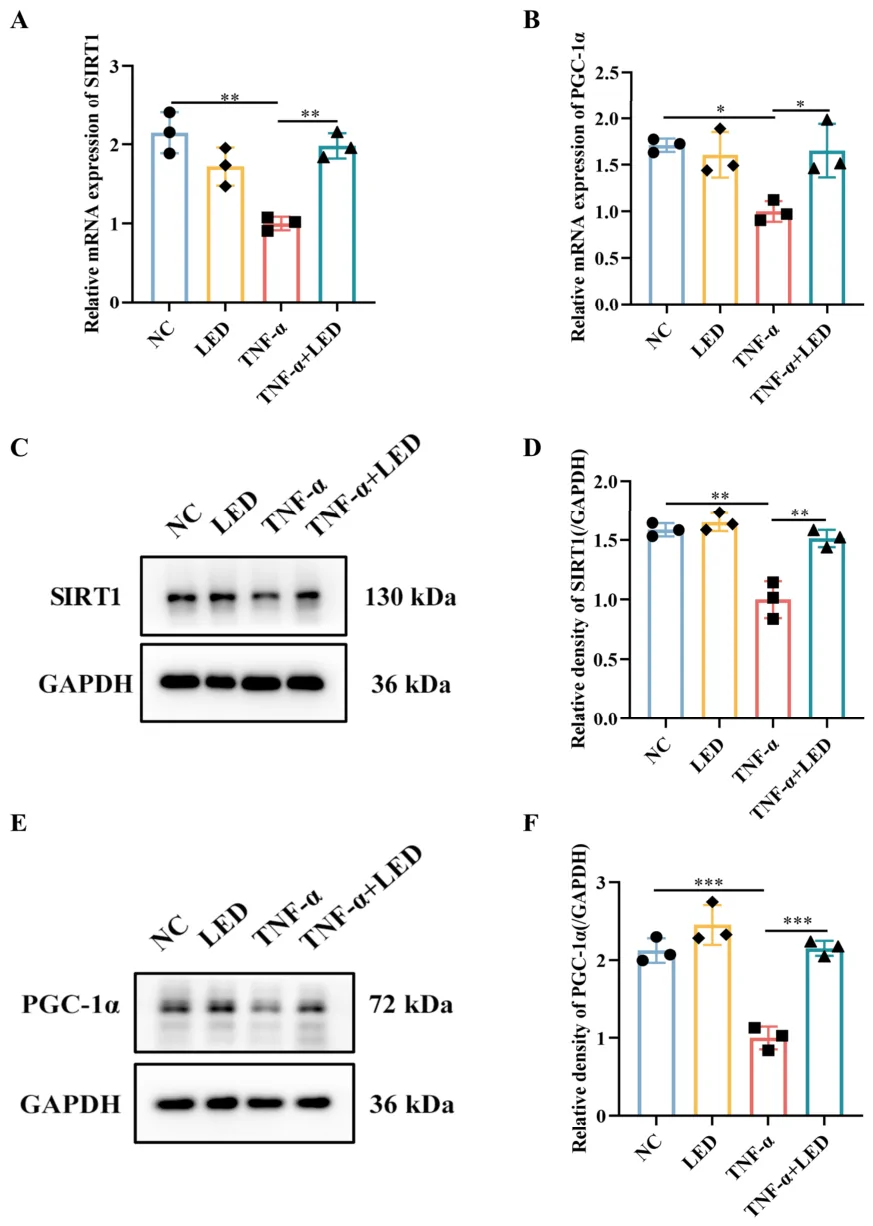
LED irradiation at 630 nm restores SIRT1 and PGC-1α expression in TNF-α-stimulated C28/I2 cells. (**A**,**B**) RT-qPCR analysis of SIRT1 and PGC-1α mRNA expression. (**C**–**F**) Representative Western blot and quantification of SIRT1 and PGC-1α protein abundance. GAPDH was used as the loading control. Data are presented as mean ± SD and were analyzed using one-way ANOVA followed by Tukey’s multiple comparisons test. Circles, diamonds, squares, and triangles represent individual data points for the NC, LED, TNF-α, and TNF-α + LED groups, respectively. *n* = 3, * *p* < 0.05, ** *p* < 0.01, *** *p* < 0.001; ns, not significant.

Because the transcriptomic results in C28/I2 cells suggested that LED treatment was associated with changes in mitochondrial homeostasis-related pathways, we next examined the expression of SIRT1 and PGC-1α. RT-qPCR analysis showed that TNF-α stimulation decreased SIRT1 and PGC-1α mRNA expression in C28/I2 cells, whereas 630 nm LED irradiation restored their expression (Figure 4A,B). Western blotting showed a similar feature at the protein level. TNF-α treatment reduced SIRT1 and PGC-1α protein abundance, whereas LED treatment reversed these reductions (Figure 4C–F). These results show that the attenuation of TNF-α-induced molecular responses by LED in C28/I2 cells is accompanied by restoration of SIRT1 and PGC-1α at both mRNA and protein levels.

### 2.5. LED Irradiation at 630 nm Attenuates Oxidative Stress and Mitochondrial Dysfunction and Alters Autophagy-Associated Proteins in TNF-α-Stimulated C28/I2 Cells

**Figure 5:**
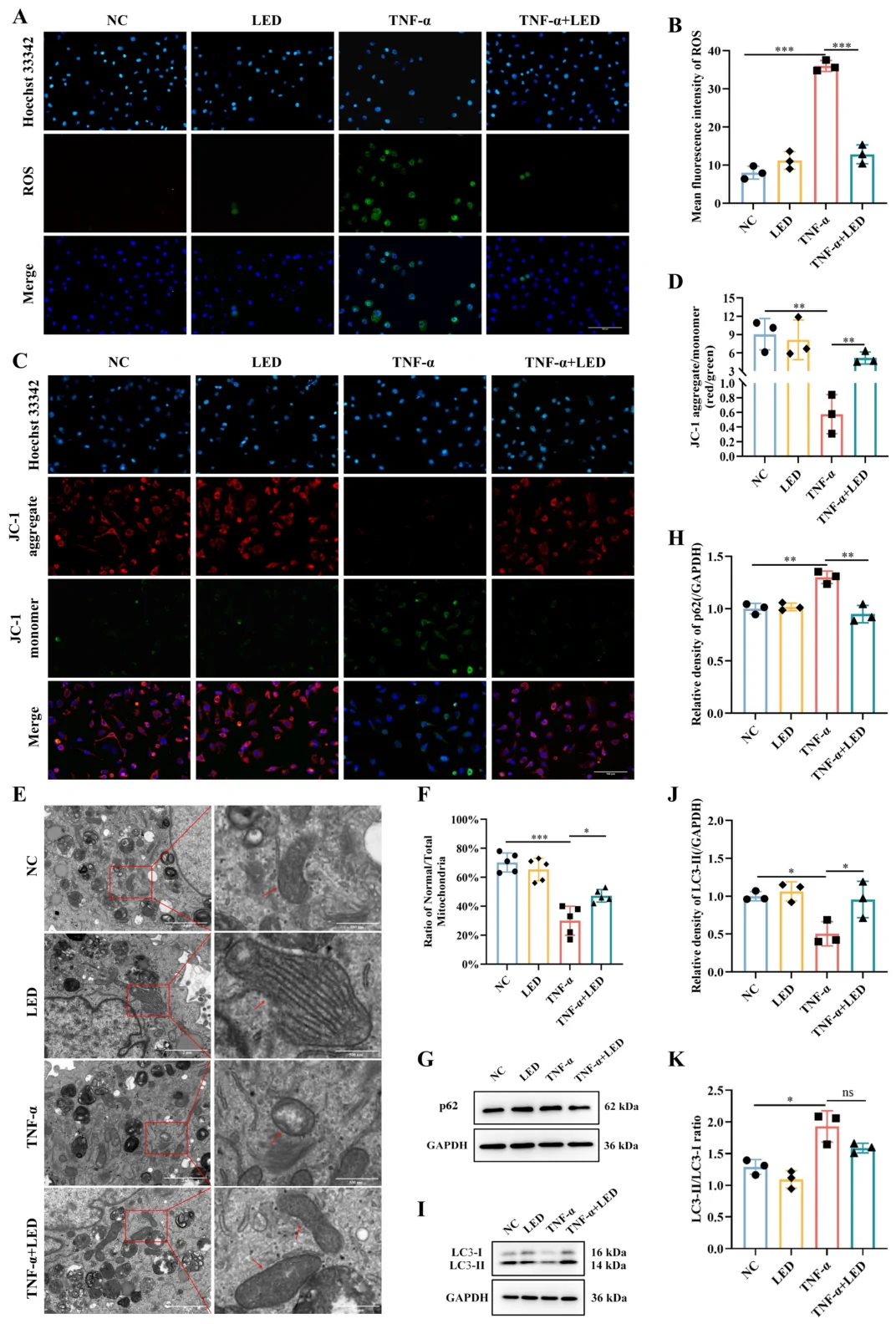
LED irradiation at 630 nm attenuates oxidative stress and mitochondrial dysfunction and alters autophagy-associated proteins in TNF-α-stimulated C28/I2 cells (**A**–**D**) Representative fluorescence images and quantification of intracellular ROS levels and mitochondrial membrane potential. Nuclei were counterstained with Hoechst 33342. Scale bar, 100 μm. (**E**) Representative transmission electron microscopy images showing mitochondrial ultrastructure. Scale bars, 2 μm and 500 nm. Red arrows indicate representative mitochondria in different groups, and the red box indicates the region shown at higher magnification. (**F**) Quantitative analysis of the percentage of morphologically normal mitochondria from TEM images. (**G**–**K**) Representative Western blots and quantification of p62, LC3-II, and the LC3-II/LC3-I ratio. GAPDH was used as the loading control. Data are presented as mean ± SD and were analyzed using one-way ANOVA followed by Tukey’s multiple comparisons test. Circles, diamonds, squares, and triangles represent individual data points for the NC, LED, TNF-α, and TNF-α + LED groups, respectively. *n* = 3 for panels (**A**–**D**,**G**–**K**). For panel (**F**), *n* = 5 independent biological samples per group. * *p* < 0.05, ** *p* < 0.01, *** *p* < 0.001; ns, not significant.

To further determine whether LED treatment was associated with improved mitochondrial function, we assessed ROS levels, mitochondrial membrane potential, and mitochondrial ultrastructure. ROS staining showed low intracellular ROS levels in the control and LED-alone groups, whereas TNF-α markedly increased ROS accumulation. LED treatment reduced ROS fluorescence intensity in TNF-α-stimulated C28/I2 cells (Figure 5A,B). JC-1 staining further showed that TNF-α treatment decreased mitochondrial membrane potential, as indicated by a lower JC-1 red/green fluorescence ratio. LED irradiation restored this ratio, suggesting preserved mitochondrial membrane potential under inflammatory stress (Figure 5C,D). Transmission electron microscopy revealed that TNF-α treatment caused mitochondrial swelling and disorganized cristae. Quantitative analysis showed that the percentage of normal mitochondria was significantly decreased in the TNF-α group compared to the control. Conversely, LED treatment ameliorated these mitochondrial damages and partially restored the proportion of normal mitochondria (Figure 5E,F). We next assessed autophagy-related proteins. TNF-α treatment increased p62 abundance and decreased LC3-II expression. Although the LC3-II/LC3-I ratio was increased after TNF-α stimulation, this increase coincided with a marked reduction in LC3-I, indicating that the elevated ratio mainly reflected lower LC3-I abundance rather than enhanced LC3-II expression. Compared with TNF-α treatment alone, 630 nm LED irradiation reduced p62 abundance, partially recovered LC3-II expression, and produced a numerical decrease in the LC3-II/LC3-I ratio that did not reach statistical significance relative to the TNF-α group (Figure 5G–K). Thus, LED irradiation was associated with partial normalization of autophagy-related protein patterns under inflammatory stimulation; however, because steady-state p62 and LC3-II measurements reflect both autophagosome formation and degradation, these data do not directly resolve the direction of autophagic flux. Collectively, these findings suggest that LED-mediated attenuation of TNF-α-induced inflammatory gene expression is accompanied by reduced ROS levels, preserved mitochondrial membrane potential, improved mitochondrial ultrastructure, and altered autophagy-associated protein levels.

### 2.6. Effects of 630 nm LED Irradiation on SIRT1 and PGC-1α Immunostaining in Joint Tissues of hTNF-α Transgenic Mice

**Figure 6:**
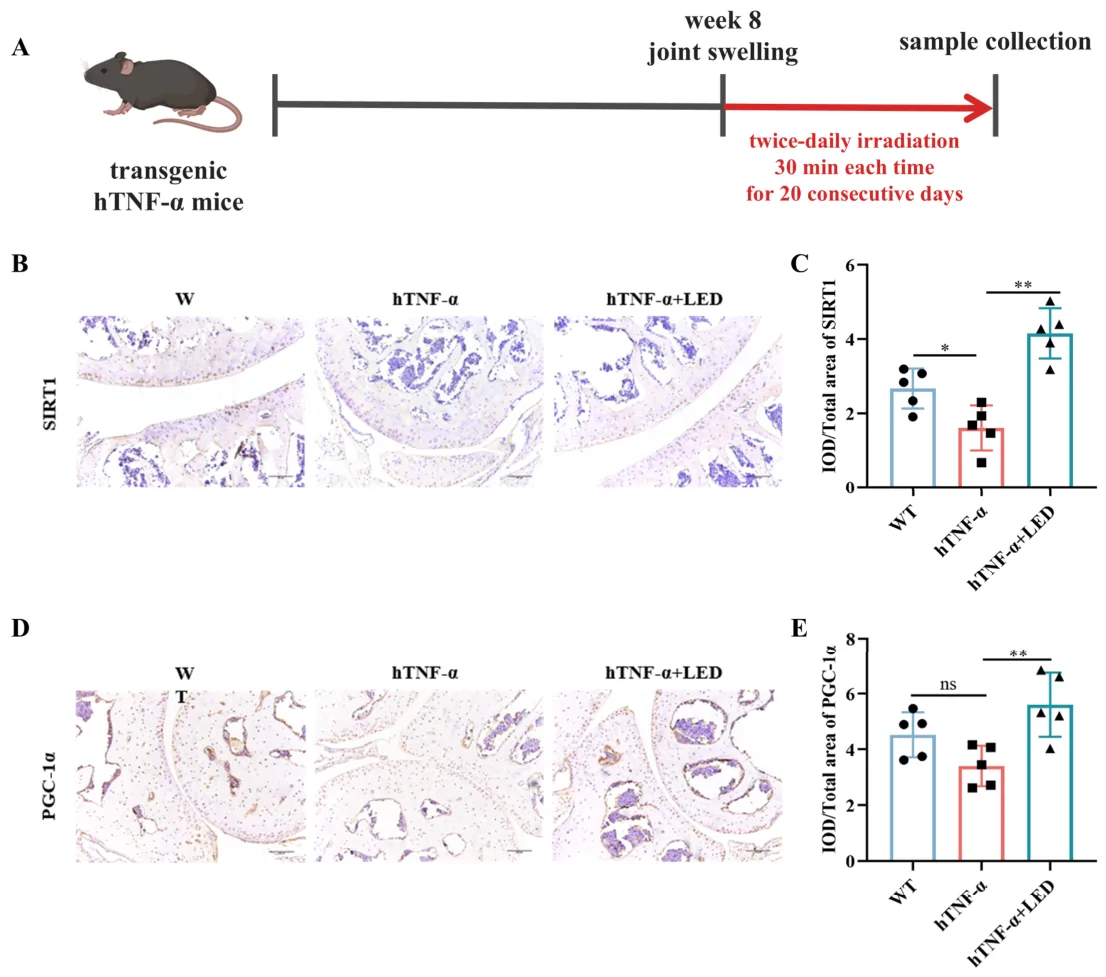
Effects of 630 nm LED irradiation on SIRT1 and PGC-1α immunostaining in joint tissues of hTNF-α transgenic mice. (**A**) Schematic overview of the 630 nm LED irradiation protocol in hTNF-α transgenic mice. (**B**–**E**) Representative immunohistochemical staining images and quantification of SIRT1 and PGC-1α expression in tissues from WT mice, hTNF-α transgenic mice, and LED-treated hTNF-α transgenic mice. Staining intensity was quantified as IOD/total area. Scale bar, 100 μm. Data are presented as mean ± SD and were analyzed using one-way ANOVA followed by Tukey’s multiple comparisons test. Circles, squares, and triangles represent individual data points for the WT, hTNF-α, and hTNF-α + LED groups, respectively. *n* = 5, * *p* < 0.05, ** *p* < 0.01; ns, not significant.

To determine whether the SIRT1/PGC-1α changes observed in vitro were also detectable in vivo, we performed immunohistochemistry in joint tissues of hTNF-α transgenic mice following the protocol shown in Figure 6A. SIRT1 staining was lower in hTNF-α transgenic mice than in WT mice and increased after 630 nm LED treatment (Figure 6B,C). PGC-1α was not significantly different between WT and hTNF-α mice; LED treatment increased joint PGC-1α in the transgenic group (Figure 6D,E). These in vivo findings show that 630 nm LED irradiation increased SIRT1 and PGC-1α immunostaining in joint tissues of hTNF-α transgenic mice.

## 3. Discussion

Our findings indicate that 630 nm LED attenuates TNF-α-induced inflammatory and senescence-associated responses in MH7A synovial cells and C28/I2 chondrocytes in association with changes in mitochondrial homeostasis-related markers. Based on transcriptomic analysis, in vitro cell experiments, and in vivo tissue validation, we propose that persistent TNF-α exposure drives joint tissue cells into a pathological state in which inflammatory senescence and mitochondrial dysfunction reinforce each other. This view is consistent with the concept that chronic, low-grade inflammation accelerates tissue aging through oxidative stress, metabolic dysregulation, and organelle dysfunction [20]. In this work, TNF-α triggered senescence-associated changes in joint tissue cells, acting as more than an isolated inflammatory stimulus. From the perspective of the biological actions of PBMT, the present findings support its potential use as a physical intervention for inflammatory and senescence-associated cellular responses. Previous studies have shown that the biological effects of red and near-infrared light are generally associated with mitochondrial photoreactions and the regulation of redox homeostasis, which can further influence cellular stress adaptation and transcriptional responses [21,22]. In this study, LED treatment alone did not induce an obvious inflammatory response. However, under TNF-α-induced stress, LED treatment exerted a protective effect. These observations suggest that the main significance of LED treatment lies in regulating stress adaptation in damaged cells, rather than non-specifically enhancing cellular activity. This interpretation is also consistent with the biphasic dose–response pattern of PBMT [23].

In the present study, we used a TNF-α-stimulated joint-associated cell model and hTNF-α transgenic mice for in vivo validation. These models were used to mimic joint cell stress and senescence driven by persistent inflammatory signaling [24,25]. Previous studies have shown that inflammation is closely linked to cellular senescence, and that both processes contribute to joint tissue degeneration and functional impairment [7]. TNF-α is strongly associated with the chronic inflammatory joint microenvironment. Previous studies have shown that TNF-α can induce senescence-associated changes in human chondrocytes, including ROS accumulation, G0/G1 phase arrest, decreased telomerase activity, and activation of p53/p21 signaling [24]. In vivo, hTNF-α transgenic mice develop chronic inflammatory joint pathology due to sustained expression of human TNF-α. Therefore, this model is commonly used to study TNF-driven tissue damage [25].

The attenuation of senescence-associated responses was reflected in reduced expression of p16, p21, and SASP-related mediators, including IL-6, IL-1beta, IL-8, MMP-3, and MMP-13. Cellular senescence is not simply a state of growth arrest. It also involves a secretory program that can amplify local tissue inflammation and disrupt extracellular matrix homeostasis [26,27]. Accordingly, the downregulation of these mediators is consistent with reduced senescence-associated paracrine damage and catabolic signaling in the joint microenvironment, in line with the concept that senescent cells are active contributors to age-related tissue dysfunction and represent modifiable therapeutic targets [28,29,30]. Our data therefore indicate an attenuation of senescence-associated markers.

These changes in senescence-associated molecular responses were accompanied by alterations in mitochondrial homeostasis-related features. TNF-α induced higher ROS levels, loss of mitochondrial membrane potential, and disruption of mitochondrial ultrastructure. Damaged mitochondria can promote cellular senescence through oxidative stress, disturbed energy metabolism, and pro-inflammatory signaling [31,32,33]; conversely, mitochondrial dysfunction can drive senescence-associated secretory activity, whereas relief of mitochondrial stress attenuates pro-senescent features [34,35,36]. Mitochondrial quality control has been recognized as an important determinant of cellular senescence [37]. LED treatment reduced ROS accumulation, preserved mitochondrial membrane potential, and improved mitochondrial structure in this study. These findings suggest that the attenuation of senescence-associated changes is accompanied by relief of the mitochondrial damage-driven inflammatory state, supporting mitochondrial homeostasis as a therapeutic target in inflammation-driven joint pathology.

Our earlier study using an essentially comparable regimen reported mitochondrial depolarization and the induction of apoptosis in MH7A synoviocytes [38], whereas PBMT preserved membrane potential in the TNF-α-stimulated C28/I2 chondrocytes examined in the present study. This divergence may reflect cell-type-specific metabolic demands. In the inflamed rheumatoid synovium, FLS undergo a metabolic reprogramming characterized by enhanced glycolytic flux. This reprogramming, induced by inflammatory cytokines such as TNF-α via GLUT1/HIF-1α, sustains their hyperproliferative and apoptosis-resistant phenotype [39,40]. In these setting, mitochondrial depolarization-driven apoptosis may reduce the hyperplastic FLS burden and, by extension, constrain synovial overgrowth. This possibility aligns with the therapeutic goal of depleting arthritogenic FLS. Chondrocytes likewise primarily depend on glycolysis to meet ATP demands in their hypoxic niche. The two cell types are not distinguished by whether they use glycolysis: chondrocyte glycolysis is a constitutive adaptation to the hypoxic niche, whereas the high glycolytic flux of rheumatoid FLS is an activation-induced phenotype, and healthy FLS retain substantial oxidative phosphorylation and metabolic flexibility [40,41]. In chondrocytes, mitochondrial oxidative phosphorylation remains indispensable for redox balance and metabolic adaptation, but this function is impaired under inflammatory conditions [41]. Consequently, preserving mitochondrial membrane potential is critical to avoid chondrocyte metabolic exhaustion and catabolic breakdown. Furthermore, the biphasic dose–response of PBMT suggests that identical influences can elicit divergent outcomes depending on the baseline redox state and metabolic context of the cell [42]. Therefore, synoviocytes and chondrocytes showed opposite membrane potential responses to 630 nm LED irradiation.

Concomitant with these mitochondrial changes, we observed altered expression of autophagy-related proteins together with restored SIRT1/PGC-1α expression. Autophagy maintains cellular homeostasis by removing damaged proteins and abnormal organelles. It is also a key quality control process that limits the accumulation of dysfunctional mitochondria and prevents sustained activation of stress signals. When autophagy is impaired, the clearance of abnormal mitochondria is reduced, which can further enhance oxidative stress, inflammatory signaling, and cellular senescence responses [43]. Previous studies have shown that activation of autophagy under experimental conditions can protect joint tissues, and that FoxO-dependent autophagy programs also help maintain chondrocyte homeostasis [44,45]. In this research, LED treatment reduced p62 expression, whereas the LC3-II/LC3-I ratio did not differ significantly between the TNF-α and TNF-α + LED groups at the single time point examined. The LC3-II/LC3-I ratio only reflects the relative conversion of LC3-I to LC3-II. It is also affected by the expression levels of both LC3-I and LC3-II. Therefore, this ratio should not be used alone to determine changes in autophagy activity [46].

The recovery of SIRT1 and PGC-1α expression suggests that LED may reactivate metabolic adaptation and mitochondrial renewal programs. SIRT1 acts as a NAD^+^-dependent deacetylase that bridges aging-related inflammation, cellular senescence, and mitochondrial biogenesis. It exerts these effects largely by deacetylating and activating PGC-1α [47,48]. In the context of chronic joint inflammation, TNF-α is known to promote SIRT1 inactivation in chondrocytes, and this loss of SIRT1 activity is intimately linked to chondrocyte senescence [49,50]. Therefore, the PBMT-induced restoration of this axis is particularly relevant as it may counteract the TNF-α-driven metabolic deterioration.

In addition to its role in mitochondrial renewal, SIRT1 may also mediate the anti-inflammatory effects of 630 nm LED. SIRT1 deacetylates NF-κB p65 at Lys310, thereby terminating NF-κB-driven transcription [51]. This mechanism is consistent with the reduced SASP secretion observed in TNF-α-challenged chondrocytes and with corresponding findings in synoviocytes [15]. Through PGC-1α and p65, SIRT1 may therefore coordinate mitochondrial renewal with inflammatory control. SIRT1 has also been reported to inactivate the PI3K/AKT/mTOR pathway, restoring autophagic flux [52]. This may facilitate the clearance of damaged mitochondria. In our earlier work, we observed changes in TRPV4 and BMP4 in MH7A synoviocytes [18,53]. TRPV4 is reported to drive Ca^2+^/CaMKII–DRP1-mediated mitochondrial injury in chondrocytes [54], whereas BMP4 is reported to restore PINK1-dependent mitophagy [55]. These studies did not examine 630 nm LED. Nevertheless, these findings link pathways related to TRPV4 and BMP4 to chondrocyte mitochondrial integrity, thereby indirectly supporting the proposed SIRT1/PGC-1α-mediated protective response to 630 nm LED.

This study has several limitations. Our findings mainly demonstrate an association between the restoration of SIRT1/PGC-1α expression and the protective effect of LED treatment. Future studies using SIRT1 or PGC-1α knockdown, overexpression, or pharmacological inhibition are needed to further clarify the causal relationship. While we observed improved mitochondrial homeostasis, mitochondrial-specific superoxide was not directly measured using probes such as MitoSOX, and mitochondrial biogenesis was not independently confirmed using markers such as TFAM expression or mtDNA copy number. Furthermore, the use of the immortalized cell lines MH7A and C28/I2 may compromise canonical senescence pathways. Consequently, future validation in primary joint cells using classical markers such as SA-β-gal staining, p16/p21 quantification, and DNA damage foci is essential to verify this phenotype. In addition, our inferences regarding autophagy-related changes rely on the steady-state levels of p62 and LC3-II; further evaluation using lysosomal inhibitors or the mRFP-GFP-LC3 reporter system will be needed to better assess autophagic flux. Finally, the in vivo analysis was limited to SIRT1 and PGC-1α immunostaining, lacking comprehensive evaluation of joint histopathology, local inflammatory mediators, and in vivo senescence markers.

In summary, this study places 630 nm LED irradiation within the framework of inflammatory senescence and mitochondrial homeostasis. These findings suggest that 630 nm LED irradiation serves as a promising non-invasive intervention strategy for mitigating TNF-α-induced inflammation-associated cellular senescence responses, accompanied by the modulation of mitochondrial homeostasis.

## 4. Materials and Methods

### 4.1. Public Transcriptomic Dataset Acquisition and Analysis of Chronically Inflamed Synovial Tissues

To characterize transcriptional features of synovial tissues under chronic inflammatory conditions, two public synovial tissue datasets, GSE77298 and GSE55235, were obtained from the Gene Expression Omnibus (GEO) database. Synovial tissues from inflammatory disease samples were defined as the chronic inflammation group, and synovial tissues from healthy donors were defined as the control group. Expression matrices were converted to official gene symbols according to the corresponding platform annotation files. When multiple probes mapped to the same gene, the probe with the highest mean expression was retained. Based on the normalized expression matrices, principal component analysis was performed using the WeiShengXin online platform (https://www.bioinformatics.com.cn/, accessed on 13 November 2025) to assess the overall distribution of gene expression profiles between the two groups. Differential expression analysis was then performed separately for the two datasets using SangerBox (http://sangerbox.com/, accessed on 13 November 2025), with *p* < 0.05 and |log_2_FC| > 1 as the screening criteria. The identified differentially expressed genes (DEGs) were further used for Gene Ontology (GO) functional annotation and Kyoto Encyclopedia of Genes and Genomes (KEGG) pathway enrichment analysis to identify inflammation-related biological functions and signaling pathways. To further identify biological processes that were consistently altered in chronically inflamed synovial tissues, gene set enrichment analysis was performed on whole-genome ranked gene lists using GSEA software version 4.3.3. GO biological process enrichment results derived from DEGs were then integrated with the GSEA results to identify commonly enriched functional pathways under chronic inflammatory conditions and to determine their enrichment direction. Pathway enrichment results were visualized using R software version 4.4.1. Detailed information on the datasets is provided in Table 1.

### 4.2. mRNA Sequencing Analysis of MH7A and C28/I2 Cells

Total RNA extraction and mRNA sequencing were performed by Tianhao Biotechnology (Shanghai, China) and Majorbio Biotechnology (Shanghai, China) for MH7A and C28/I2 cells, respectively. Both cell lines were subjected to identical conditions, comprising four groups: negative control, LED irradiation, TNF-α treatment, and TNF-α + LED treatment. MH7A samples (*n* = 3 per group) were sequenced on the Illumina HiSeq 2500 platform (Illumina, San Diego, CA, USA; paired-end 150 bp reads). C28/I2 samples were sequenced on the Illumina NovaSeq platform (Illumina, San Diego, CA, USA; paired-end 150 bp reads); although four biological replicates were initially processed, stringent quality control resulted in the retention of three valid replicates for the LED irradiation and TNF-α treatment groups, and four replicates for the remaining groups (*n* = 3–4 per group). DEGs were identified using *p* < 0.05 and |log_2_FC| > 1 as the screening criteria. Volcano plots were generated using SangerBox (http://sangerbox.com/, accessed on 16 May 2026). Gene set enrichment analysis was performed using GSEA software version 4.3.3 to determine whether predefined gene sets were coordinately enriched between treatment groups. Pathways were selected using the following criteria: *p* < 0.05, |NES| > 1, and FDR < 0.25. Enrichment results were visualized using R software. The MH7A transcriptome was analyzed for TNF-α-induced inflammatory and senescence-associated responses, whereas the C28/I2 transcriptome was leveraged for PBMT-related mitochondrial and autophagy alterations.

### 4.3. Cell Culture and LED Irradiation

A 630 nm LED device (CPYL3213–630, Truwin Optoelectronic Medical, Beijing, China) was used for light irradiation. For the in vitro experiments, LED irradiation was delivered at a power density of 44.06 mW/cm^2^ and an energy density of 39.6 J/cm^2^ per session. These irradiation parameters were selected based on our previous studies [18]. The human chondrocyte cell line C28/I2 and the human fibroblast-like synoviocyte cell line MH7A were purchased from Qingqi Biotechnology (Shanghai, China) and Beina Biotechnology (Beijing, China), respectively. Cells were cultured in complete DMEM medium (Gibco, Waltham, MA, USA) supplemented with 10% fetal bovine serum (Gibco, Waltham, MA, USA) and 1% penicillin/streptomycin (Beyotime, Shanghai, China). Cells were maintained at 37 °C in an incubator with 5% CO_2_. After 12 h of plating, cells were divided into four groups: negative control, LED irradiation, TNF-α treatment, and TNF-α + LED treatment. To mimic the inflammatory microenvironment, cells were stimulated with 10 ng/mL TNF-α (PeproTech, Cranbury, NJ, USA). Following 6 h of TNF-α stimulation, the LED irradiation groups were exposed to 630 nm irradiation for five 15-min sessions, with a 1.5-h interval between consecutive sessions. Cells were harvested 30 min after the final exposure for subsequent assays. Non-irradiated groups were wrapped with aluminum foil to avoid light exposure and maintained under the same room temperature conditions.

### 4.4. Animal Experiments and LED Irradiation

To evaluate the effects of LED irradiation under chronic inflammatory conditions in vivo, hTNF-α transgenic mice on a C57BL/6J background (6–8 weeks old, 18–22 g; Cyagen catalog No. C001250) and age-matched WT C57BL/6J mice were obtained from Cyagen (Suzhou, China). Fifteen male mice were used in total: five WT mice served as the WT control, and ten hTNF-α transgenic mice were randomly divided into the hTNF-α model group (*n* = 5) and the hTNF-α + LED treatment group (*n* = 5). Mice were housed under standard specific pathogen-free conditions with a 12 h light/dark cycle. The animal room was maintained at 22 ± 2 °C and 60 ± 5% relative humidity. During the treatment period, mice from three groups were placed in identical transparent acrylic cages; however, the first two groups did not receive 630 nm LED irradiation, whereas the paws of mice in the hTNF-α + LED group were exposed to 630 nm LED irradiation twice daily for 30 min per session for 20 consecutive days. LED irradiation was delivered at a power density of 8.2 mW/cm^2^ and an energy density of 14.76 J/cm^2^ per session. These irradiation parameters were selected based on our previous studies [18]. On day 21, the mice were euthanized, and paw tissues were collected for subsequent analyses. All animal experiments were approved by the Animal Ethics and Welfare Committee of Harbin Medical University under approval number HMUIRB2023003.

### 4.5. Real-Time Quantitative Polymerase Chain Reaction

Total RNA was extracted from cells using TRIzol reagent (Invitrogen, Carlsbad, CA, USA). A total of 2 μg of RNA was reverse-transcribed into cDNA using the HiFiScript cDNA Synthesis Kit (Cwbio, Beijing, China). Real-time quantitative PCR was performed using the LightCycler^®^ 96 system (Roche Applied Science, Mannheim, Germany). Gene expression was expressed as cycle threshold (Ct) values, and the relative mRNA expression of target genes in each experimental group was calculated using the 2^−ΔΔCt^ method relative to the control group. Primer sequences are listed in Table 2.

### 4.6. Western Blotting

Pretreated cells were washed three times with phosphate-buffered saline (PBS) and lysed on ice for 30 min in RIPA lysis buffer (Beyotime, Shanghai, China) supplemented with phosphatase inhibitors (Meilunbio, Dalian, China). The cell lysates were scraped, collected, and then centrifuged at 12,000 rpm for 15 min. The supernatants were collected, and protein concentrations were measured using a BCA assay kit (Beyotime, Shanghai, China). Equal amounts of protein were separated by SDS-PAGE and transferred onto PVDF membranes (Vazyme, Nanjing, China). The membranes were blocked with 5% non-fat milk for 2 h and incubated with primary antibodies overnight at 4 °C. The next day, the membranes were washed with TBST and incubated with secondary antibodies for 1 h at room temperature. After another washing, the antibody-bound signals were detected using super sensitive ECL luminescence reagent (Meilunbio, Dalian, China). Images were analyzed using ImageJ software version 1.52a (NIH, Bethesda, MD, USA). Detailed antibody information is provided in Table 3.

### 4.7. Immunohistochemical Staining

Mouse paw tissues were decalcified, embedded in paraffin, and sectioned into 5 μm sections. After deparaffinization, rehydration, and antigen retrieval, the sections were incubated with 3% hydrogen peroxide for 10 min at room temperature to block endogenous peroxidase activity. The sections were then blocked with goat serum diluted in PBS (Boster, Pleasanton, CA, USA) for 1 h to reduce nonspecific antibody binding. After blocking, the sections were incubated overnight at 4 °C with anti-PGC-1α (1:200, Proteintech, Wuhan, China) and anti-SIRT1 (1:200, Proteintech, Wuhan, China). The next day, the sections were washed with PBS and stained using 3,3′-diaminobenzidine (DAB) peroxidase substrate (Zsbio, Beijing, China). All sections were observed under a light microscope, and images were captured for further analysis.

### 4.8. Transmission Electron Microscopy

Transmission electron microscopy (TEM) was used to observe mitochondrial ultrastructure and morphological changes. Fresh cell samples were fixed in electron microscopy fixative containing 2.5% glutaraldehyde at room temperature for 30 min and then stored at 4 °C. The samples were sent to Servicebio (Wuhan, China) for embedding and sectioning, and were cut into 60–80 nm ultrathin sections. Mitochondrial images were observed and captured using an HT7800 transmission electron microscope (Hitachi, Tokyo, Japan). For quantitative analysis, mitochondria with intact outer membranes and organized cristae were defined as morphologically normal. The percentage of morphologically normal mitochondria was quantified for each group. For each biological sample, values from multiple randomly selected image fields were averaged to yield a single measurement per sample.

### 4.9. Intracellular ROS Detection

Intracellular ROS levels in live cells were detected using a DCFH-DA fluorescent probe (Solarbio, Beijing, China). After LED irradiation, the culture medium was removed, and the cells were washed with PBS. The cells were then incubated with the diluted DCFH-DA working solution at 37 °C for 30 min in the dark, followed by two washes with serum-free medium to remove the unbound probe. Nuclei were counterstained with Hoechst 33342 prior to imaging. Intracellular DCF fluorescence signals were observed and captured using a fluorescence microscope (Leica, Wetzlar, Germany). The mean DCF fluorescence intensity of the entire field of view was quantified using ImageJ, with background subtraction. To ensure comparability, the initial cell seeding density and imaging parameters including exposure time and gain were strictly standardized across all groups.

### 4.10. JC-1 Staining

Mitochondrial membrane potential was assessed using a JC-1 staining kit (Solarbio, Beijing, China). After LED irradiation, cells were washed with PBS and incubated with a 1:1 mixture of complete culture medium and JC-1 working solution for 30 min at 37 °C in the dark. Following incubation, cells were washed twice with JC-1 buffer and imaged in fresh complete medium. Red and green fluorescence signals were captured using a fluorescence microscope (Leica, Wetzlar, Germany) under consistent exposure and gain settings. ImageJ was then used to quantify the intensities, and the red/green ratio was calculated for statistical analysis.

### 4.11. Statistical Analysis

All experiments were independently repeated at least three times, and data are presented as mean ± SD. Statistical analyses were performed using GraphPad Prism 9.5. Comparisons among multiple groups were performed using one-way ANOVA followed by Tukey’s multiple comparisons test. Statistical significance was set at *p* < 0.05.

## Figures and Tables

**Table 1:** The synovial RNA expression data of patients and healthy control obtained from the GEO database.

Dataset	Platform	Sample Type	NC	Inflammatory Samples
GSE77298	GPL570	Synovial tissue	7	16
GSE55235	GPL96	Synovial tissue	10	10

**Table 2:** Primers for RT-qPCR.

Primer	Sequence
Human-SIRT1-F	TGAAGTTGACCTCCTCATTGTTATT
Human-SIRT1-R	CTTTCTGGTGAACTTGAGTCTTCTG
Human-PGC-1α-F	CCATGTAGAGATTCTGTGTCACCAC
Human-PGC-1α-R	TCAGACTCTCGCTTCTCATACTCTC
Human-IL-1β-F	AATAACAAGCTGGAATTTGAGTCTG
Human-IL-1β-R	TGCATGGTGAAGTCAGTTATATCCT
Human-IL-6-F	GGTCCAGTTGCCTTCTCCCT
Human-IL-6-R	TTACATGTCTCCTTTCTCAGGGC
Human-IL-8-F	GACATACTCCAAACCTTTCCACC
Human-IL-8-R	AAACTTCTCCACAACCCTCTGC
Human-MMP-3-F	TCAGAACCTTTCCTGGCATC
Human-MMP-3-R	TCAACAGCAGAATCAACAGCA
Human-MMP-13-F	CCTTGATGCCATTACCAGTCTCC
Human-MMP-13-R	AAACAGCTCCGCATCAACCTGC
Human-p16-F	AGCAGCATGGAGCCTTCGG
Human-p16-R	GCCCATCATCATGACCTGGATCG
Human-p21-F	GATGAGTTGGGAGGAGGCAG
Human-p21-R	GCGAGGCACAAGGGTACAAG
Human-β-actin-F	CACTCTTCCAGCCTTCCTTCC
Human-β-actin-R	GGACTCGTCATACTCCTGCTTG

**Table 3:** Primary and secondary antibodies and dilution ratio.

Target	Company	Cat. No.	Dilution Ratio
SIRT1	Proteintech	13161-1-AP	1:2000
PGC-1α	Proteintech	66369-1-Ig	1:1000
p62	Abmart	T55546F	1:1000
LC3B	Abmart	T55992F	1:2000
MMP-3	PTM Biolabs	PTM-6130	1:2000
GAPDH	Immunoway	YM3029	1:10,000
Vinculin	Proteintech	26520-1-AP	1:10,000
anti-mouse HRP secondary antibody	Immunoway	RS0001	1:5000
anti-rabbit HRP secondary antibody	Immunoway	RS0002	1:5000

## Data Availability

The public datasets analyzed in this study are available from the GEO database under accession numbers GSE77298 and GSE55235. The remaining data supporting the findings of this study are available from the corresponding author upon reasonable request.
